# The MR1/MAIT cell axis reduces phagocytosis and dystrophic neurites in Alzheimer’s disease

**DOI:** 10.1002/alz.087706

**Published:** 2025-01-03

**Authors:** Season K Johnson, Samantha Ackley, Jalyn Warren, Randy R Brutkiewicz

**Affiliations:** ^1^ Stark Neurosciences Research Institute, Indiana University School of Medicine, Indianapolis, IN USA; ^2^ Stark Neuroscience Research Institute, Indianapolis, IN USA

## Abstract

**Background:**

Plaques are a hallmark feature of Alzheimer’s disease (AD). We found the loss of mucosal‐associated invariant T (MAIT) cells and its antigen‐presenting molecule MR1 caused a delay in plaque pathology development in AD mouse models. However, it remains unknown how this axis is impacting microglial response and dystrophic neurites. This study aims to understand the impact of MAIT cells and microglial MR1 in AD.

**Method:**

Brain tissue from various ages of 5XFAD mice and those that are MR1‐deficient (MR1KO), was analyzed for the presence of MAIT cells. Methoxy‐X04 was used to analyze the phagocytic capacity of microglia ± MR1. Immunofluorescent microscopic analysis of dystrophic neurites in the brain was performed with antibodies against microglia, Aβ, Lamp1, Ubiquitin, and nAPP.

**Result:**

Injection of Methoxy‐X04 in 5XFAD and 5XFAD/MR1KO mice revealed reduced levels of Methoxy‐X04 uptake in CD11b^+^CD45^low^ cells in the MR1 KO group (P < 0.05). However, this remained unaltered in the CD11b^+^CD45^high^ cells. In the 5XFAD/MR1 KO group there was reduced expression of LAMP1, Ubiquitin, and nAPP in the hippocampus at 8 months compared to 5XFAD mice (P < 0.001). In the cortex only nAPP remained reduced in the 5XFAD/MR1 KO mice (P < 0.001).

**Conclusion:**

The loss of MR1 and MAIT cells reduced the phagocytic capacity of microglia and dystrophic neurite formation in the hippocampus. Our data indicate a potential detrimental role for MR1 and/or MAIT cells in AD pathology. Understanding this axis of the innate immune system could provide new clues as to the overall role of innate immunity in AD and its potential as a therapeutic target in AD.

